# Smoking Cessation Smartphone App for Nondaily Smoking With Telephone Onboarding: Proof-of-Concept Randomized Controlled Trial

**DOI:** 10.2196/53971

**Published:** 2025-01-15

**Authors:** Bettina B Hoeppner, Kaitlyn R Siegel, Allison E Futter, Diadora Finley-Abboud, Alivia C Williamson, Christopher W Kahler, Elyse R Park, Susanne S Hoeppner

**Affiliations:** 1 Health through Flourishing (HtF) program Department of Psychiatry Massachusetts General Hospital Boston, MA United States; 2 Department of Psychiatry, Massachusetts General Hospital Harvard Medical School Boston, MA United States; 3 Center for Alcohol and Addiction Studies Department of Behavioral and Social Sciences Brown University School of Public Health Providence, RI United States; 4 Health Promotion and Resiliency Intervention Research Center Mongan Institute/Department of Psychiatry Massachusetts General Hospital Boston, MA United States

**Keywords:** mobile health, mHealth, smoking cessation, nondaily smoking, smartphone, smoking, positive psychology, mobile phone

## Abstract

**Background:**

Nondaily smoking is a widespread and increasingly prevalent pattern of use. To date, no effective treatment approach for nondaily smoking has been identified.

**Objective:**

This study aimed to conduct an unblinded randomized controlled trial to evaluate proof-of-concept markers of the Smiling instead of Smoking (SiS) app, a smartphone app for smoking cessation, designed specifically for people who smoke less than daily, within the framework of positive psychology.

**Methods:**

Overall, 226 adults who smoke less than daily were recruited on the web and asked to undertake a quit attempt while using assigned smoking cessation support materials. Participants were randomly assigned to 1 of 3 materials: the SiS smartphone app, the National Cancer Institute’s smartphone app QuitGuide (QG), or the National Cancer Institute’s smoking cessation brochure, “Clearing the Air” (CtA). All participants engaged in a 15-minute scripted onboarding phone call and were introduced to their support materials to use for the next 7 weeks. Follow-up self-assessment web surveys were sent 2, 6, 12, and 24 weeks after participants’ initially chosen quit date (ie, 1 week after onboarding). The primary outcome for this study was self-efficacy to remain abstinent from smoking at treatment end. Secondary outcomes assessed treatment acceptability, treatment feasibility (eg, number of days of app use, time spent using app, and use of smoking cessation strategies), and secondary proof-of-concept efficacy outcomes (eg, positive affect, craving, and attitudes toward smoking). Smoking outcomes (ie, 30-day point prevalence abstinence and smoking reduction) were also assessed.

**Results:**

Results indicated a significant effect of treatment on the primary outcome, where SiS participants (n=80) reported higher self-efficacy to abstain from smoking at the end of treatment than the 2 control groups (QG: n=75; *P*=.02; Cohen *d*=0.40 and CtA: n=71; *P*=.007; Cohen *d*=0.50). This effect was also significant on both self-efficacy subscales (ie, internal cues and external cues) with effect sizes ranging from Cohen *d*=0.34 to 0.50 across the pairwise comparisons. The SiS app group also reported lower craving (QG: *P*=.005; Cohen *d*=–0.57 and CtA: *P*=.005; Cohen *d*=–0.57) and higher positive affect than QG (QG: *P*=.01; Cohen *d*=0.44 and CtA: *P*=.05; Cohen *d*=0.38); attitudes toward smoking were largely similar across groups. Treatment acceptability was comparable across groups (*P* values for all groups >.05; Cohen *d* range 0.06-0.23). Treatment feasibility measures indicated that participants used the SiS app on 33 out of 49 days, for 35 to 40 minutes per week, resulting in greater use of smoking cessation strategies than QG (QG: *P*=.04; Cohen *d*=0.38 and CtA: *P*=.16; Cohen *d*=0.24).

**Conclusions:**

These findings provide strong evidence for the conceptual underpinnings of the SiS app, and thereby provide compelling justification for conducting a large-scale randomized controlled trial that can test the effectiveness of the SiS app on smoking cessation.

**Trial Registration:**

ClinicalTrials.gov NCT04672239; https://clinicaltrials.gov/study/NCT04672239

**International Registered Report Identifier (IRRID):**

RR2-10.2196/40867

## Introduction

### Background

Nondaily smoking is an increasingly prevalent pattern of smoking, which despite perceptions to the contrary, results in substantial health detriments. Currently, 25% of all US adults who smoke do so less frequently than daily [1]. This prevalence has increased by 27% in the last decade [1]. Formerly believed to be a transient pattern of smoking [2,3], research has established that nondaily smoking is a persistent pattern [4-8]. Nondaily smoking is more prevalent among Black and Latinx populations [9-11] and is increasingly prevalent among people with serious mental health issues [12]. People who smoke less than daily and have never smoked daily are younger than those who smoke daily or have previously smoked daily [13]. More than 10 years ago, nondaily smoking was highlighted as an important public health issue [14]. This call to action has produced compelling evidence on the substantial negative impact of nondaily smoking on health [15], observed for smoking as few as 6 to 10 cigarettes per month [13].

Despite the prevalence of nondaily smoking, the US Clinical Practice Guidelines for smoking cessation [16] offer no guidance on how to support people who smoke less than daily in smoking cessation due to a lack of evidence for efficacious approaches. People who smoke less than daily do not view themselves as “smokers” who need “treatment” and thus are challenging to engage in traditional smoking cessation treatments [17-20]. They do, however, report high motivation to quit smoking, more so than people who smoke daily [3,17,18], which manifests in more recent and planned cessation efforts [18,21-23]. To date, only 2 trials have tested interventions for nondaily smoking cessation. Both focused on pharmacological treatments (ie, nicotine replacement therapy) and both failed to show efficacy in achieving smoking abstinence [24,25]. This lack of efficacy is in line with perceptions by people who smoke less than daily that withdrawal is not a barrier to their smoking cessation [26,27]. Behavioral intervention approaches may be more effective. Smartphone apps are a highly sought after source of behavioral support, as demonstrated by >33 million downloads that smoking cessation apps have generated [28]. Moreover, this technology-facilitated approach invokes less treatment resistance among people who smoke less than daily than more traditional smoking cessation treatments [20].

### Using the Smiling instead of Smoking Smartphone App to Support People Who Smoke Less Than Daily in Quitting Smoking

Building on research that found that people who smoke less than daily prioritized having positive self-identity and wellness [26], our team built a smartphone app that focused on fostering the experience of positive emotions, the Smiling instead of Smoking (SiS) app [29,30]. As detailed in the protocol paper matching this outcome report [31], the treatment approach of the SiS app was inspired by the development of positive psychotherapy for smoking cessation [32,33]. Typically, during smoking cessation, positive affect decreases temporally, following a U-shaped trajectory consistent with a withdrawal effect [34]. However, research has shown that having high positive affect is beneficial to quitting in that it is related to increased self-efficacy to abstain from smoking [35], decreased desire to smoke [36,37], and greater readiness to process self-relevant health information [38]. While positive psychotherapy for smoking cessation was originally developed for daily smokers, research with people who smoke less than daily has highlighted the impact of positive affect on reducing craving [36]. As many other constructs related to smoking cessation are less salient to people who smoke less than daily [26], we chose “fostering positive affect” as the therapeutic goal of the SiS app.

To develop the SiS app, we used an iterative, staged process, consisting of 3 studies. This paper reports on the outcomes of the third study (NCT04672239). The 2 prior studies demonstrated the app’s ability to engage people who smoke less than daily when onboarded in-person and when onboarded remotely [30,39]. User experiences by participants in both studies were used to further adapt and develop the app, as described previously [31,39]. In both studies, within-person changes were observed in line with our conceptual model [30,39]. Moreover, in a feature-level analysis of data obtained during the second study (NCT03951766), the number of days participants used the app significantly predicted 30-day point prevalence abstinence (PPA) at the end of treatment and 6-month follow-up [40]. Further analyses indicated that this effect was primarily due to interacting with the positive psychology components of the SiS app, rather than the app’s tools dedicated to smoking cessation guidance.

### Objectives

In this paper, we present the outcomes of the first randomized trial testing the SiS app, using a parallel, unblinded, randomized design. As described in the protocol paper [31], this study is a proof-of-concept randomized controlled trial (RCT), where we targeted increased self-efficacy by treatment end as a proximal, conceptual proof-of-concept indicator of treatment efficacy. We chose self-efficacy as our primary, proximal outcome variable because of its prominence in theoretical models of health behavior change [41-44] and because of prior demonstrable effects. Namely, a large-scale efficacy trial of an SMS text messaging intervention for smoking cessation identified self-efficacy as the primary mediator of treatment on conferring benefit for smoking cessation [45].

In this trial, we tested the SiS app against 2 control conditions to allow us to choose the more rigorous control condition for a subsequent, large-scale efficacy RCT. Both control conditions use smoking cessation materials developed and disseminated by the National Cancer Institute (NCI): the NCI’s smartphone app QuitGuide (QG) and the NCI’s smoking cessation brochure “Clearing the Air” (CtA), respectively. Secondary goals of this proof-of-concept RCT were to compare treatment acceptability and feasibility across groups, describe smoking cessation outcomes, and test for differences on secondary proof-of-concept efficacy outcomes. In line with our conceptual model, we chose positive affect, craving, and attitudes toward smoking as these secondary conceptual markers of efficacy.

## Methods

### Participants

Participants were adults who smoke less than daily and were interested in using support materials to help them quit smoking (recruitment period: February 25, 2021, to June 29, 2022). Study recruitment information was displayed on Craigslist, Facebook, Reddit, Smokefree.gov, ClinicalTrials.gov, a study recruitment website at Massachusetts General Hospital, and a study specific website, and was shown to people interacting with study recruitment websites (ie, Clinical Connection and Wayturn). To be eligible, participants had to be above the age of 18 years, own an Android or iPhone smartphone, smoke cigarettes at least weekly but no more than 25 out of the past 30 days, have a lifetime history of having smoked >100 cigarettes, be willing to make a quit attempt as part of the study, and currently reside in the United States.

For this proof-of-concept RCT, we selected a sample size that would allow us to detect group differences on our primary outcome variable (ie, self-efficacy) [31]. A large RCT testing a mobile health intervention for smoking cessation found group differences of Cohen *d*=0.66 on self-efficacy 1 month after quitting [45]. We conservatively chose to power the trial to detect Cohen *d*=0.50, as our primary end point was further out (ie, 6 weeks after quitting).

### Procedure

The study was conducted entirely remotely. Participants were recruited on the web nationwide within the United States, using the tagline “are you smoking nondaily and want to quit?.” Interested participants were phone screened, where they learned more about the study (ie, that the study involves randomization, that some but not all groups would use a smartphone app, and that there would be web-based survey spanning 6 months). Following this phone screen, interested people were emailed a screening survey. This survey contained check items (eg, “Please indicate ‘strongly agree’”) to assess participants’ ability to successfully interact with the web-based surveying platform, REDCap (Research Electronic Data Capture; Vanderbilt University) [46]. Screening participants who completed this survey were invited to an enrollment phone call and asked to provide contact information for family or friends who would be available to the study staff to help contact participants, in case the participants changed their contact information during the course of the study. Participants were advised that the enrollment phone call needed to be scheduled to occur 1 week before their quit date, but that they could choose their quit day, and the enrollment call would be scheduled to accommodate their chosen quit day. If their chosen quit date occurred before the study closed for enrollment, their preference was accommodated. On average, 19 (SD 15) days elapsed between participants completing the screening survey and their chosen quit day.

During the enrollment phone call, the study fact sheet was reviewed, smoking status was reconfirmed via self-report, and participants provided verbal consent to enroll in the study. Following consent, while on the call, study staff emailed participants a link to download their assigned app (for both apps, links to the GooglePlay and iStore listings were sent; both apps were freely available to the public at no cost) or the PDF for CtA, as chosen by randomization using a 1:1:1 allocation; randomization occurred via randomization sheet, with staff looking up a participant’s group assignment within the 24 hours before the enrollment visit to prepare for the onboarding. Staff then engaged participants in a scripted 15-minute onboarding dialogue based on their assigned treatment condition. Staff did not specify which treatment condition was the intervention of treatment versus the control conditions, but rather presented each assigned smoking cessation material as potentially useful to help support smoking cessation. The onboarding script length was matched between randomized groups. This scripted dialogue systematically led participants through their assigned smoking cessation materials, with study staff asking participants to read out loud the text displayed by their assigned app or brochure and asking participants to share answers to questions their smoking materials asked them to think about.

At the end of onboarding, study staff instructed participants to use their assigned app or brochure for a period of 7 weeks (1 week before quitting and 6 weeks after quitting) and to complete follow-up surveys on the web 2, 6, 12, and 24 weeks after their initially chosen quit date. Participants were told that they could change their quit date as needed, but that this initially chosen quit date would be the anchor date for surveying.

In total, we screened 1268 individuals over phone. Of these, 41.4% (525/1268) of the individuals were found ineligible during the phone screening (primarily due to not smoking less than daily; 497/525, 94.7%), and 15.3% (194/1268) of the individuals decided against the study (most commonly due to having lost interest in the study; 56/194, 28.9%) or not liking the study as described (22/194, 11.3%), but also due to study logistics, such as needing to provide their social security number for payment by check (36/194, 18.6%) or having to wait for checks to be mailed (26/194, 13.4%). The remaining 43.3% (549/1268) of the individuals were emailed the screening survey. Of these, 175 (31.9%) chose not to complete the survey, 86 (15.7%) failed the check items embedded in the survey, and 2 (0.4%) completed the survey after the study had closed enrollment. The remaining 52.1% (286/549) of the individuals were invited to the enrollment phone call. Of these, 28 (9.8%) participants did not show up for the enrollment phone call, 20 (7%) participants decided against the study at this point, and 1 (0.3%) participant could not be reached to schedule the enrollment. The remaining 82.9% (237/289) of the individuals started the enrollment phone call. During this phone call, 2.8% (8/237) of the individuals were found ineligible (ie, n=5 did not smoke less than daily, n=2 did not want to quit smoking, and n=1 did not reside in the United States). The remaining 96.6% (229/237) of the individuals were enrolled and proceeded to onboarding. During onboarding, after having learned about their group assignment, 3 participants (one in each treatment group) decided against the study, resulting in a final sample size of 226 participants who were enrolled and successfully onboarded to their smoking cessation materials.

Survey responses were obtained from 95.1% (215/226), 89.4% (202/226), 82.3% (186/226), and 81.9% (185/226) of the participants at 2, 6, 12, and 24 weeks after the initially chosen quit day, respectively; these included participants who only completed partial surveys (12/226, 5.3%; 8/226, 3.5%; 7/226, 3.1%; and 9/226, 4%, respectively) and participants who completed the survey but incorrectly responded to 2+ check items (6/226, 2.7%; 6/226, 2.7%; 7/226, 3.1%; and 2/226, 0.9%, respectively). Obtaining survey responses did not differ between groups (all *P*>.07 at all assessments).

### Treatment Condition: SiS Smartphone App

**Figure 1:**
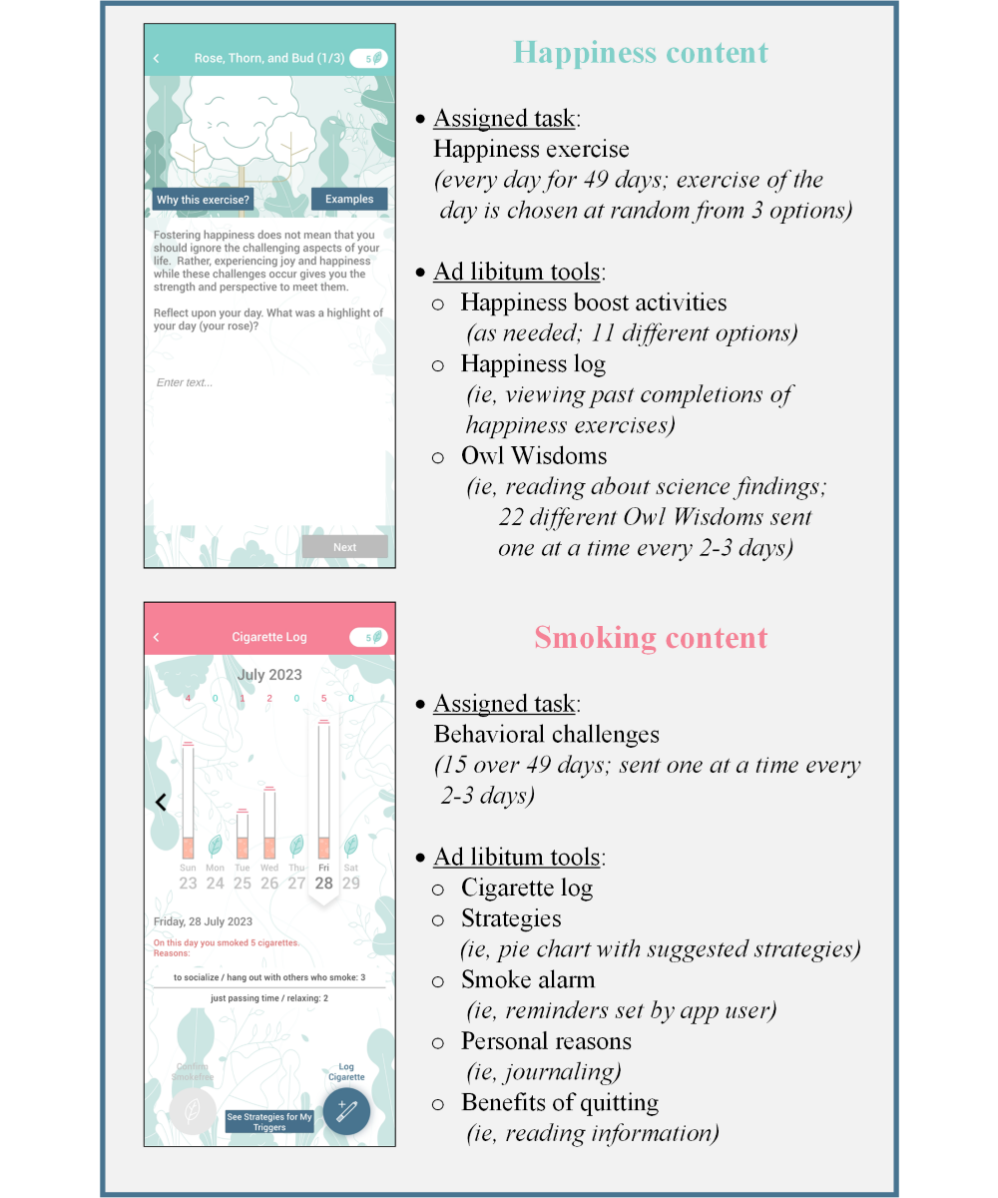
Summary of the content provided in the Smiling instead of Smoking (SiS) app.

Participants in the treatment condition were asked to use the SiS app every day for 7 weeks. As described in more detail elsewhere [31], the SiS app provides smoking cessation tools within the framework of positive psychology. Thus, app users were asked to engage in activities that foster positive affect and in activities that focus on smoking cessation (Figure 1). To enhance prescriptive clarity [40], a tool called “Today’s Tasks” listed all tasks to be completed that day; clicking on the task brought the app user directly to that task in the app.

The positive psychology content was derived from positive psychology findings, as summarized previously [47]. App users were asked to complete a positive psychology habit-building exercise every day to develop a habit of noticing and savoring the positive experiences app users encountered in everyday life. For moments of low positive affect, the SiS app offered a variety of happiness boost activities. The rationale for engaging in positive affect fostering activities was reinforced throughout the app: the overall rationale was provided in a section called “why work on happiness”; “why” buttons were linked to all exercises, which provided information about the rationale for each specific exercise; and push notifications, called “Owl Wisdoms,” were sent every 2-3 days to provide positive psychology science findings relevant to the tasks the app assigned at that time.

The smoking cessation content built directly on the materials provided by the NCI’s Smokefree.gov resources, which are in line with United States Clinical Practice Guidelines [16]. The smoking cessation content consisted of tools to engage app users in tracking their cigarette use, understanding their triggers for smoking, learning about benefits of quitting smoking, and reflecting on personal reasons to quit smoking. Guidance to engage with these tools was provided via time-anchored push notifications, called “Behavioral Challenges,” which asked app users to complete a specific task within the app on that specific day.

During the course of this trial, the SiS app, as hosted on hospital servers, experienced three downtimes, lasting from 1 hour to 2 days, where the app did not record its use, but users could still interact with the app in its intended way. The downtimes were noted by the research team at Massachusetts General Hospital; the app programmers (ie, PreviewLabs Inc) then implemented the needed changes in the programming to overcome outages. No changes were made to the overall functionality of the app during the duration of this study.

### Control Condition 1: NCI QG Smartphone App

Participants in control condition 1 were asked to use the QG app every day for 7 weeks. As described in more detail elsewhere [31], the QG app provided many tools analogous to the smoking cessation content provided in the SiS app, just designed differently. Specifically, functionality exists to set the quit date, track mood and craving, read about best practices for quitting smoking, log cigarettes, see graphs about smoking patterns based on one’s cigarette log, set reminders to stay smoke free (time- and location-based), and receive tips in response to reporting craving or negative affect. QG is frequently used as a comparison app in smartphone app smoking cessation studies [28,48-53] because it controls for time, attention, and modality of delivery of smoking cessation support.

### Control Condition 2: NCI CtA Brochure

Participants in control condition 2 were asked to use the CtA brochure every day for 7 weeks. As described in more detail elsewhere [31], this 36-page brochure [54] provided analogous smoking cessation information as the QG app. It is composed of both text and worksheets to engage the reader in reflecting on reasons to quit, learning about the benefits of quitting, logging smoking and its triggers, and formulating strategies for dealing with triggers. This booklet has been used as a “treatment as usual” comparison condition in past RCTs evaluating phone-based and other mHealth smoking cessation technologies [55-58].

### Measures

Two sources of data were collected: self-report answers to web-based surveys and passively recorded app use data. The primary end point for this study was treatment end, which occurred 6 weeks after the originally chosen quit date.

#### Primary Outcome

The primary outcome for this proof-of-concept RCT was self-efficacy to abstain from smoking, as measured using the Self-Efficacy Questionnaire, assessed at baseline, and 2, 6, and 12 weeks after the initially chosen quit day. This 12-item scale asks participants to rate their confidence on a 0 to 100 slider scale in their ability to abstain from smoking when facing internal stimuli (eg, feeling depressed) and external stimuli (eg, being with people who smoke). Scale scores are reported as mean scores (range 0-100), where higher scores indicate greater self-efficacy to abstain from smoking. The primary outcome variable was the total Self-Efficacy Questionnaire score. Subscale scores (ie, internal stimuli and external stimuli) were also calculated to provide more nuanced insight and are presented on the same mean score scale. The internal consistency (Cronbach α) of the total score ranged from 0.89 at baseline to 0.95 at the 6-month follow-up, for the internal subscale from 0.86 at baseline to 0.95 at the 6-month follow-up, and for the external subscale from 0.80 at week 2 to 0.91 at the 6-month follow-up in this sample.

#### Secondary Outcomes: Treatment Acceptability

Treatment acceptability was assessed at treatment end using the Client Satisfaction Questionnaire (CSQ) [59] to assess satisfaction with the received smoking cessation support, the System Usability Scale (SUS) [60] to assess acceptability of the technology used to provide smoking cessation support, and 2 single item measures to assess overall ratings of treatment likeability and satisfaction. The CSQ scores are reported as total scores that can range from 0 to 27, where higher scores indicate higher client satisfaction; the internal consistency of CSQ scores was 0.95 in this sample. The SUS scale scores are transformed to a 0 to 100 range, where higher scores mean greater system usability. Interpreted on a letter grading system, SUS scores in the range from 78.9 to 80.7 correspond to an A-, 80.8 to 84.0 to an A, and 84.1 to 100 to an A+ [61]. The internal consistency of SUS scale scores was 0.88 in this study. Both single item measures were rated on 5-point Likert scales ranging from 1=“I strongly disliked using the app” to 5=“I strongly liked using the app” for likeability and from 1=“very unsatisfied” to 5=“very satisfied” for satisfaction.

#### Secondary Outcomes: Treatment Feasibility

Treatment acceptability was assessed at multiple time points. For participants randomized to use apps, acceptability was measured by their actual engagement with their assigned apps (ie, number of days participants used their assigned apps) and their subjective appraisal of how much (in minutes per week) they used their assigned apps at midtreatment (week –1 to week 2 after the quit date) and at the end of treatment (week 3 to 6 after the quit date). All participants, regardless of whether they were assigned a smartphone app, were asked to reflect at treatment end on how much time they spent applying content learned through the smoking cessation support materials (in minutes per week), to report on recommended smoking cessation strategies they used, and to rate the perceived impact of their assigned smoking cessation treatment. Smoking cessation strategies used were evaluated with an 8-item survey rated on 5-point Likert scales ranging from 1=“strongly disagree” to 5=“strongly agree,” and aggregate scores were presented as mean scores, where higher scores indicate a higher use of multiple cessation strategies. The internal consistency of this Use of Smoking Cessation Strategies scale was 0.80 in this sample. Because the SiS app uses a positive psychology approach to smoking cessation, we also included items that addressed positive psychology approaches to smoking cessation in this list (eg, “I focused on feeling as happy as possible while I was quitting smoking” and “I focused on the good things that happened each day”), as used in prior research on positive psychotherapy for smoking [33]. These items were summarized into a similar mean score of Use of Positive Psychology Strategies (same range and interpretation as the Use of Smoking Cessation Strategies) and had an internal consistency of 0.88 in this sample. The perceived impact of assigned smoking cessation treatments was evaluated with a 17-item survey, as used in prior studies on the SiS app [30,39], (eg, [The assigned treatment] “...made me think that it was worthwhile for me to quit.”, “...gave me the feeling I could get trusted advice at any time.”) rated on a 5-point Likert scale ranging from 1=“strongly disagree” to 5=“strongly agree.” Aggregate scores were presented as mean scores, where higher scores indicated a higher perceived impact of the assigned treatment on participants’ smoking cessation support. The internal consistency of the Perceived Impact scale was 0.95 in this sample.

#### Secondary Outcomes: Exploratory Treatment Effectiveness Outcomes

While this trial was not powered to detect differences in smoking cessation, we did assess smoking cessation outcomes for descriptive purposes. Specifically, we assessed 30-day PPA, as self-reported smoking status 6, 12, and 24 weeks after the initially chosen quit day. Participants who did not report on smoking status were presumed to be smoking. We also assessed smoking reduction (ie, the difference in cigarettes smoked in the past week, assessed at baseline vs the end of treatment).

#### Secondary Outcomes: Secondary Proof-of-Concept Markers

In addition to the primary outcome, we assessed additional constructs in line with our conceptual model. Specifically, we used the positive affect subscale of the Positive and Negative Affect Schedule [62] to assess positive affect within the past week and the Brief Questionnaire of Smoking Urges (Brief-QSU) [63] to assess in the moment craving. The 10-item positive affect subscale of the Positive and Negative Affect Schedule is reported as a total score that can range from 10 to 50, with higher scores representing higher levels of positive affect; the internal consistency of this scale ranged from 0.91 at week 2 to 0.94 at the 6-month follow-up. The 10-item Brief-QSU scale is reported as a total score that can range from 7 to 70, with higher scores indicating greater cigarette craving. In this sample, the internal consistency of the Brief-QSU ranged from 0.92 at baseline to 0.96 at the 6-month follow-up. To assess attitudes toward smoking, we used the 3 subscales of the Attitudes Toward Smoking [64] scale, and the Decisional Balance Inventory for Smoking short form (DCB-SF) [65]. The Attitudes Toward Smoking subscales have different numbers of items, so we present each subscale score as a mean score with a possible range from 1=“strongly disagree” to 5=“strongly agree,” so that higher scores indicate stronger agreement with statements about the Adverse Effects of Smoking (10 items), the Psychoactive Benefits of Smoking (4 items), and the Pleasure of Smoking (4 items) for the respective subscales. The internal consistencies of all 3 subscales ranged from 0.82 for the Psychoactive Benefits of Smoking at baseline to 0.94 for the Adverse Effects of Smoking at the 3-month follow-up. The DCB-SF is a 6-item scale that is rated on 0 to 100 slider scales. Three DCB-SF items evaluate the positive smoking expectancies (pros) and 3 items evaluate the negative smoking expectancies (cons); item scores are averaged within each subscale for a score range of 0 to 100, where higher scores indicate greater agreement with the pros or cons of smoking, respectively. The internal consistency of the subscales ranged from 0.74 to 0.85 for pros and 0.68 to 0.78 for cons across the assessments.

#### Participant Descriptors: Demographics, Smoking Characteristics, and Clinical Characteristics

At baseline, participants reported on demographic information, smoking characteristics, and clinical characteristics. Most items were stand-alone multiple-choice items. We used validated scales to assess nicotine dependence, depression severity, anxiety severity, and capacity to experience pleasure. Specifically, we used the Fagerström Test for Cigarette Dependence [66], the Center of Epidemiologic Studies Depression Scale [67], the Generalized Anxiety Disorder Screener [68], and the Snaith-Hamilton Pleasure Scale [69], respectively. For cigarette dependence, Fagerström Test for Cigarette Dependence total scores can range from 0 to 10, where higher scores indicate greater dependence. To summarize clinical characteristics, we used a Center of Epidemiologic Studies Depression Scale cutoff score of ≥10 to indicate the risk of depression [67], a Generalized Anxiety Disorder Screener cutoff score of ≥10 to indicate moderate anxiety [68], and a Snaith-Hamilton Pleasure Scale cutoff score of >2 to indicate anhedonia [69], an inability to feel pleasure.

### Analytic Strategy

#### Data Preparation

Before analysis, we reviewed all check items participants completed as part of their web-based surveys. Data were set to missing for a specific scale if the participant incorrectly responded to the check item embedded in that scale. Data were set to missing for the entire time point, if the participant responded incorrectly to ≥2 check items. Scale scores were then calculated; if an item was left blank, the scale score was calculated using the average of the remaining items (after accounting for reverse coding, as applicable), so long as ≥80% of the items were completed.

#### Analysis of Primary Outcome

To test if randomized group assignment was significantly related to treatment outcome, we used a generalized linear mixed model, where repeated observations per person were modeled using an unstructured covariance matrix. Predictors included in the model were GROUP (ie, SiS vs QG vs CtA), TIME (ie, baseline, week 2, week 6, week 12, and week 24), and the GROUP*TIME interaction effect. A contrast statement was used to derive the test statistic for the GROUP comparison at week 6, our primary endpoint, given the overall longitudinal model. The contrast statement tested the null hypothesis that SiS=QG=CtA. If this null hypothesis was rejected (*P*<.05) for the primary outcome, we followed this up with pair-wise follow-up tests to compare the SiS app treatment to each of the specific control groups (ie, SiS vs QG, SiS vs CtA); for secondary outcomes, we provided these secondary pairwise comparisons for all outcomes, regardless of statistical significance of the overall effect. Between-group effect sizes were calculated using Cohen *d*, where effect sizes are interpreted such that 0.2 is small, 0.5 is medium, and 0.8 is large [70]. Details about the significance tests and pairwise comparisons are available in Tables S1 and S2 in Multimedia Appendix 1.

#### Analysis of Secondary Outcomes

The same analytic strategy was used for secondary outcomes, but adjusted for fewer assessments (ie, no TIME predictor, if outcome was only assessed once), and the distribution of the variable of interest (ie, logit model used for the binary outcome 30-day point prevalence smoking abstinence). Some outcomes (including change in past week cigarettes, time spent applying content, app use, and app use) were heavily skewed to the right; for these outcomes, we used the nonparametric Wilcoxon rank sum test to analyze group differences (Table S3 in Multimedia Appendix 1). Effect sizes for these outcomes were reported as *r*=*Z*/sqrt(*N*), where *Z* is the test statistic from the Wilcoxon rank sum test and *N* is the total number of observations (ie, participants), and which are interpreted such that 0.1 is small, 0.3 is medium, and 0.5 is large [70]. Effect sizes for the logit models are presented as odds ratios with 95% CIs. Because these secondary outcomes cover distinctly different domains and are interpreted marginally to give a fuller picture of the participant experience, we did not correct for multiple testing [71]. We calculated effect sizes to provide insight into the relative strength of these effects. All analyses were performed using SAS System for Windows (version 9.4; IBM Corp).

### Ethical Considerations

The study was approved by the Mass General Brigham institutional review board (2020P003466) and registered on ClinicalTrials.gov (NCT04672239). All participants provided informed consent; specifically, they provided verbal consent after reviewing the study fact sheet and engaging in a true or false knowledge test of the content of the study fact sheet in conversation with study staff. Surveys were collected via Health Insurance Portability and Accountability Act compliant technology (ie, REDCap), which was only accessible to staff trained in the conduct of research. Smartphone data were stored on password protected Mass General Brigham servers and linked to survey data via study-generated app ID number. Participants received US $25 for completed surveys or US $10 for incomplete surveys or surveys with failed check items. They received US $50 for the week 6 survey (end of treatment), which was longer than the other surveys. Participants provided their social security number to enable remuneration by check.

## Results

### Participant Characteristics

**Figure 2:**
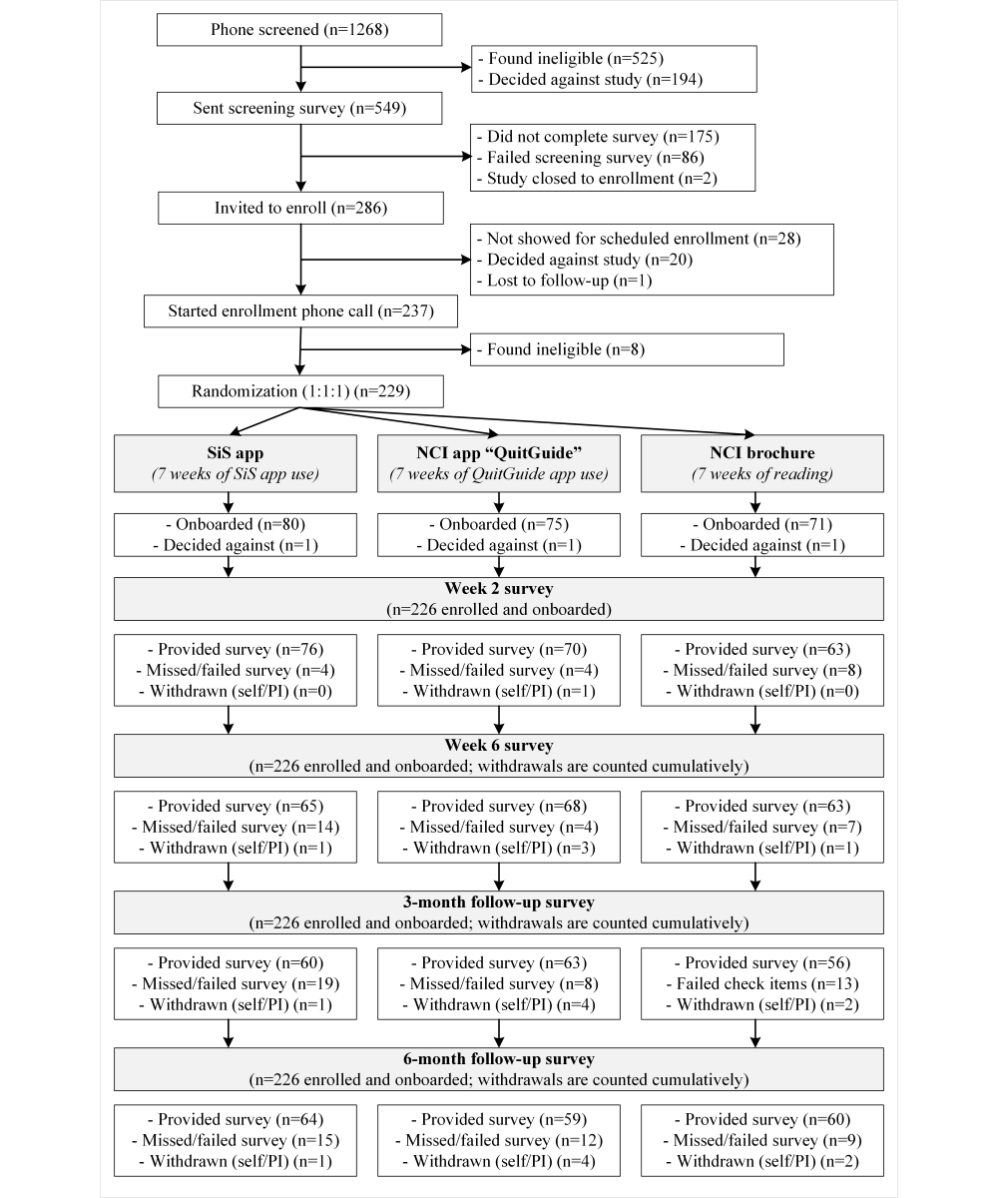
Flow of participants throughout the study from phone screening to the end of 6-month follow-up. NCI: National Cancer Institute; PI: principal investigator; SiS: Smiling instead of Smoking.

Study participants (Figure 2; CONSORT-eHEALTH checklist is provided in Multimedia Appendix 2) lived in urban, suburban, and rural areas, located in 42 out of 50 US states (Northeast region: 51/226, 22.6%; Midwest region: 31/226, 13.7%; South region: 97/226, 42.9%; West region: 47/226, 20.8%). They were largely (151/226, 66.8%) people who had smoked daily previously, who smoked an average of 3.4 (SD 2.7) cigarettes per smoking day on 14.9 (SD 4.9) days out of the past 30 days. Many had made a previous quit attempt; many had tried e-cigarettes; few were using them at the time of the study (Table 1).

**Table 1:** Baseline characteristics of study participants.

Characteristic			SiS3^a^ (n=80)	QuitGuide (n=75)	Clearing the Air (n=71)	Total (N=226)
**Demographics**						
	Age (y), mean (SD)		41.3 (11.1)	41.6 (13.1)	40.0 (12.5)	41.0 (12.2)
	**Gender, n (%)**					
		Men	28 (35)	37 (49.3)	30 (42.3)	95 (42)
		Women	51 (63.7)	36 (48)	41 (57.7)	128 (56.6)
		Nonbinary	1 (1.3)	2 (2.7)	0 (0)	3 (1.3)
	**Race, n (%)**					
		American Indian or Alaska Native	0 (0)	0 (0)	1 (1.4)	1 (0.4)
		Asian or Pacific Islander	1 (1.3)	1 (1.3)	4 (5.6)	6 (2.7)
		Black	23 (28.7)	19 (25.3)	13 (18.3)	55 (24.3)
		White	50 (62.5)	46 (61.3)	48 (67.6)	144 (63.7)
		Other or unknown	2 (2.5)	3 (4)	1 (1.4)	6 (2.7)
		More than one race	4 (5)	6 (8)	4 (5.6)	14 (6.2)
	Hispanic, n (%)		15 (18.7)	16 (21.3)	6 (8.5)	37 (16.4)
	**Education, n (%)**					
		High school or less	7 (8.7)	12 (16)	5 (7)	34 (15)
		Some college	31 (38.7)	38 (50.7)	25 (35.2)	94 (41.6)
		Bachelor of Arts, Bachelor of Science, or higher	42 (52.5)	25 (33.3)	30 (42.3)	97 (42.9)
	**Employment, n (%)**					
		Full-time	29 (36.3)	30 (40)	27 (38)	86 (38)
		Part-time	14 (17.5)	11 (14.7)	18 (25.3)	43 (19)
		Unemployed	32 (40)	33 (44)	23 (32.4)	88 (38.9)
	**Household income (US $), n (%)**					
		≤39,999	41 (51.3)	42 (56)	30 (42.3)	113 (50)
		40,000-69,999	18 (22.5)	18 (24)	22 (31)	58 (25.7)
		70,000-99,999	16 (20)	8 (10.7)	10 (14.1)	34 (15)
		≥100,000	4 (5)	7 (9.3)	8 (11.3)	19 (8.4)
	**Geographic location, n (%)**					
		Urban	39 (48.7)	34 (45.3)	27 (38)	100 (44.2)
		Suburban	29 (36.3)	24 (32)	35 (49.3)	88 (38.9)
		Rural	12 (15)	17 (22.7)	9 (12.7)	38 (16.8)
**Smoking characteristics**						
	Number of days smoked in past 30 days, mean (SD)		15.3 (5.4)	14.7 (4.8)	14.6 (4.5)	14.9 (4.9)
	Number of cigarettes smoked per smoking day, mean (SD)		3.5 (2.9)	3.0 (2.2)	3.8 (3.0)	3.4 (2.7)
	Cigarette dependence (FTCD^b^), mean (SD)		1.7 (1.9)	1.6 (1.8)	1.7 (1.6)	1.7 (1.8)
	Ever smoked daily? (yes), n (%)		54 (67.5)	53 (70.7)	44 (62)	151 (66.8)
	Ever quit before? (yes), n (%)		49 (61.3)	56 (74.7)	52 (73.2)	157 (69.5)
	Ever used E-cigarette? (yes), n (%)		46 (57.5)	41 (54.7)	46 (64.8)	133 (58.8)
	Currently using E-cigarette? (yes), n (%)		14 (17.5)	10 (13.3)	9 (12.7)	33 (14.6)
**Clinical characteristics**						
	Depressive symptoms (CES-D-10^c^) (at risk), n (%)		30 (37.5)	37 (49.3)	41 (57.7)	108 (47.8)
	Moderate to severe anxiety (GAD-7^d^) (yes), n (%)		19 (23.7)	21 (28)	19 (26.8)	59 (26.1)
	Anhedonia (SHAPS^e^) (yes), n (%)		11 (13.7)	18 (24)	17 (23.9)	46 (20.3)
	Ever diagnosed mental health condition (yes), n (%)		24 (30)	23 (30.7)	22 (31)	69 (30.5)
	**Alcohol use, past 30 days (yes), n (%)**					
		Any alcohol use	45 (56.3)	37 (49.3)	40 (56.3)	122 (54)
		Exceeded drinking guidelines	23 (28.7)	18 (24)	19 (26.8)	60 (26.5)
		Any binge drinking episode	25 (31.3)	15 (20)	18 (25.3)	58 (25.7)

^a^SiS3: version 3 of the Smiling instead of Smoking app.

^b^FTCD: Fagerström Test for Cigarette Dependence (range: 0-10, where higher scores indicate greater nicotine dependence).

^c^CES-D-10: Center of Epidemiologic Studies Depression Scale, 10-item version (yes=total score of ≥10, indicating risk of depression).

^d^GAD-7: Generalized Anxiety Disorder Screener (yes=total score of ≥10, indicating moderate or more severe anxiety).

^e^SHAPS: Snaith-Hamilton Pleasure Scale (yes=total score >2, indicating mild or more severe anhedonia, an inability to feel pleasure).

Scores on validated scales indicated presence of significant depressive symptoms for 47.8% (108/226) and moderate anxiety symptoms for 26.1% (59/226) of the participants. One-third of the participants (69/226, 30.5%) reported having been diagnosed with a mental health condition in their lifetime. Capacity to experience pleasure was in the normal range for most participants (180/226, 79.6%). A quarter of participants exceeded drinking guidelines [69] during the 30 days before the study.

### Primary Outcome: Self-Efficacy

Analyses indicated a significant difference between randomized groups at treatment end (*F*_2,198_=4.32; *P*=.01), where pair-wise follow-up tests indicated higher self-efficacy for participants randomized to SiS compared to QG (t_198.8_=2.31; *P*=.02; Cohen *d*=0.40) and CtA (t_199.7_=2.72; *P*=.007; Cohen *d*=0.50; Table 2; Tables S1 and S2 in Multimedia Appendix 1). The effect was consistent with the effect observed in subscale specific analyses, with effect sizes ranging from Cohen *d*=0.34 to 0.50 across the pairwise comparisons.

**Table 2:** Study outcomes at end of treatment (6 weeks after initially chosen quit date).

Outcome				SiS3^a^ app		QG^b^ app		CtA^c^		Effect size and *P* value, SiS3 versus																	
										QG									CtA								
										Cohen *d*^d^		Cohen r^e^			*P* value			Cohen *d*			Cohen *r*				*P* value		
**Primary outcomes**																											
	Self-efficacy (SEQ-12^f^): overall, mean (SD)		78.3 (17.3)		71.3 (17.7)		69.0 (19.8)		0.40		—^g^			.02			0.50			—				.007			
	Self-efficacy (SEQ-12): internal, mean (SD)		76.5 (20.8)		69.6 (19.4)		66.9 (21.4)		0.34		—			.03			0.45			—				.01			
	Self-efficacy (SEQ-12): external, mean (SD)		80.2 (16.4)		72.7 (19.4)		70.8 (21.3)		0.42		—			.03			0.50			—				.009			
**Secondary outcomes**																											
	**Treatment acceptability**																										
		Client satisfaction (CSQ-8^h^), mean (SD)	26.9 (5.3)		26.6 (4.5)		25.7 (5.8)		0.06		—			.75			0.23			—				.17			
		System usability (SUS^i^), mean (SD)	81.9 (17.1)		79.5 (16.0)		—		0.15		—			.41			—			—				—			
		App likability rating, mean (SD)	4.2 (1.0)		4.1 (0.9)		—		0.12		—			.50			—			—				—			
		App satisfaction rating, mean (SD)	4.3 (1.0)		4.1 (0.9)		—		0.14		—			.42			—			—				—			
	**Treatment feasibility**																										
		Use of smoking cessation strategies, mean (SD)	4.1 (0.6)		3.9 (0.6)		4.0 (0.7)		0.38		—			.04			0.24			—				.16			
		Use of positive psychology strategies, mean (SD)	4.3 (0.6)		4.0 (0.5)		4.0 (0.6)		0.55		—			.003			0.42			—				.02			
		Perceived impact on quitting, mean (SD)	4.1 (0.7)		4.0 (0.6)		3.8 (0.7)		0.23		—			.20			0.40			—				.01			
		Applying content (min/wk), median (IQR)^j^	30 (20-70)		25 (10-60)		30 (10-60)		—		0.17			.13			—			0.12				.34			
		App use^k^ (number of days used), median (IQR)^j^	33 (11-40)		26 (11-38)		—		—		0.07			.42			—			—				—			
		App use, weeks 1-3 (min/wk), median (IQR)^j^	40 (28-68)		28 (16-50)		—		—		0.23			.007			—			—				—			
		App use, weeks 4-7 (min/wk), median (IQR)^j^	35 (21-70)		23 (14-60)		—		—		0.17			.06			—			—				—			
**Exploratory outcomes (ie, secondary proof-of-concept efficacy outcomes)**																											
	**Positive affect**																										
		Positive affect (PANAS^l^), mean (SD)	37.2 (8.2)		33.4 (9.1)		34.0 (8.4)		0.44				—			.01		0.38					—				.05
	**Desire to smoke**																										
		Craving (Brief-QSU^m^), mean (SD)	14.9 (6.5)		19.7 (10.1)		19.7 (10.0)		–0.57				—			.005		–0.57					—				.005
	**Attitudes toward smoking**																										
		Adverse effects (ATS^n^), mean (SD)	4.4 (0.6)		4.4 (0.6)		4.4 (0.6)		0.03				—			.74		0.05					—				.65
		Psychoactive benefits (ATS), mean (SD)	2.6 (1.1)		2.8 (1.0)		2.9 (1.0)		–0.23				—			.17		–0.33					—				.1
		Pleasure (ATS), mean (SD)	2.3 (0.9)		2.4 (0.9)		2.7 (1.0)		–0.11				—			.44		–0.45					—				.04
		Positive expectancies (DCB-SF^o^), mean (SD)	28.6 (26.5)		36.0 (24.2)		39.1 (25.6)		–0.29				—			.12		–0.40					—				.08
		Negative expectancies (DCB-SF), mean (SD)	69.0 (27.6)		71.3 (23.0)		67.1 (24.6)		–0.09				—			.63		0.07					—				.54
	Treatment effectiveness																										
		Change in past week cigarettes (cigarettes per week), median (IQR)^j^	–5 (–12 to –1)		–6 (–11 to –3)		–7 (–13 to –4)		—		0.00			1.00			—			0.02				.97			

^a^SiS3: version 3 of the Smiling instead of Smoking app.

^b^QG: National Cancer Institute (NCI) QuitGuide app.

^c^CtA: National Cancer Institute “Clearing the Air” brochure.

^d^Cohen *d* effect size, where effects are interpreted as 0.2=small, 0.5=medium, and 0.8=large.

^e^Cohen *r* effect size, where effects are interpreted as 0.1=small, 0.3=medium, and 0.5=large.

^f^SEQ-12: Self-Efficacy Questionnaire (range 0-100, where higher scores indicate greater self-efficacy to abstain from smoking).

^g^Not applicable.

^h^CSQ-8: Client Satisfaction Questionnaire (range 0-27, where higher scores indicate higher client satisfaction).

^i^SUS: System Usability Scale (range 0-100, where higher scores mean greater app usability).

^j^Outcomes that were not normally distributed and were analyzed with Wilcoxon rank sum tests (rather than generalized linear mixed model used for all other outcomes).

^k^App use is reported for all who were onboarded (SiS3 app n=80, QuitGuide app n=75).

^l^PANAS: Positive and Negative Affect Schedule (range 10-50, where higher scores indicate higher levels of positive affect).

^m^Brief-QSU: Brief Questionnaire of Smoking Urges (range 7-70, with higher scores indicating greater cigarette craving).

^n^ATS: Attitudes Toward Smoking scale (subscale mean score range 1-5, where higher scores indicate stronger agreement with the subscale statements).

^o^DCB-SF: Decisional Balance Inventory for Smoking short form (subscale range 0-100, where higher scores indicate greater agreement with the pros and cons of smoking, respectively).

### Secondary Outcomes: Treatment Acceptability and Feasibility

Treatment acceptability was comparable across the 3 randomized groups across all indices (Table 2; Tables S1 and S2 in Multimedia Appendix 1), indicating credibility of both control groups. Technological acceptability of SiS, as measured by the SUS, was rated as an “A” on average [61]; satisfaction with the smoking cessation treatment was similarly high.

Treatment feasibility was higher for SiS compared to QG and CtA across several indices (Table 2; Tables S1 and S2 in Multimedia Appendix 1), but in different ways. In comparison to QG, SiS participants more frequently used recommended smoking cessation strategies during their treatment (Cohen *d*=0.38). Subjectively, they felt that they had spent more time with the SiS than QG app (*r*=0.23) during the first 3 weeks of treatment. This effect was not significant for the second half of treatment, or significant on the objectively measured number of days with which participants engaged with their assigned apps. In comparison to CtA, SiS participants perceived a stronger impact of their treatment on their quitting experience (Cohen *d*=0.40). In comparison to both QG and CtA, SiS participants more frequently used positive psychology strategies to help them quit smoking than QG (Cohen *d*=0.55) or CtA (Cohen *d*=0.42) participants.

### Exploratory Treatment Effectiveness Outcomes: Impact on Smoking

Self-reported 30-day PPA tended to be higher among SiS than QG or CtA participants, but not significantly so (Table 3). This trend was observable (but not significant) at both treatment end (SiS3 vs QG: *P*=.06; SiS3 vs CtA: *P*=.23) and 6-month follow-up (SiS3 vs QG: *P*=.64; SiS3 vs CtA: *P*=.55). Reductions in cigarette smoking from baseline to treatment end were comparable across treatment groups (|*r*|<0.03; Table 2).

**Table 3:** Point prevalence smoking abstinence at end of treatment and at end of follow-up.

	SiS3^a^ app (n=80), n (%)	QG^b^ app (n=75), n (%)	CtA^c^ (n=71), n (%)	OR^d^ (95% CI) SiS3 vs	
				QG	CtA
30-day PPA^e,f^ 6 weeks after quitting	32 (40)	23 (31)	18 (25)	1.5 (0.8-2.9)	2.0 (0.98-3.9)
30-day PPA 6 months after quitting	38 (48)	32 (43)	31 (44)	1.2 (0.6-2.3)	1.2 (0.6-2.2)

^a^SiS3: version 3 of the Smiling instead of Smoking app.

^b^QG: National Cancer Institute (NCI) QuitGuide app.

^c^CtA: NCI “Clearing the Air” brochure.

^d^OR: odds ratio.

^e^PPA: point prevalence abstinence.

^f^PPA is reported for all n=226 who started treatment, with missing surveys interpreted as “smoking.”

### Exploratory Outcomes: Secondary Proof-of-Concept Efficacy Outcomes

Significant group differences were found for some of the secondary proof-of-concept outcomes (Table 2; Tables S1-S3 in Multimedia Appendix 1). The strongest effect was observed for craving, where SiS participants reported significantly lower craving at treatment end than QG or CtA participants, an effect that was consistent in size across the 2 control groups (both Cohen *d*=–0.57). This effect was also stronger than the effect on self-efficacy, the primary outcome of this study. There was also a significant effect on positive affect, where SiS participants had higher positive affect at treatment end, but this effect was only significant when comparing SiS and QG participants (Cohen *d*=0.44), but not in comparing SiS and CtA participants (Cohen *d*=0.38). In terms of attitudes toward smoking, participants reported largely similar attitudes at the end of treatment (all but one|d|<0.34). The only exception was that SiS participants reported significantly lower pleasure in smoking than CtA participants at the end of treatment (Cohen *d*=–0.45). This effect was not significant compared to QG participants (Cohen *d*=–0.11).

## Discussion

### Principal Findings

We tested version 3 of the SiS app in a proof-of-concept RCT using a remote-only design and brief onboarding procedures, matched for time and content across treatment groups. We found a significant treatment effect on our primary outcome target, such that participants receiving smoking cessation support via the SiS app were more confident at treatment end in their ability to remain abstinent than participants in 2 relevant control conditions (ie, the smoking cessation app QG and the smoking cessation pamphlet CtA). Importantly, engaging with the SiS app appeared to help people who smoke less than daily face both internal and external cues to smoking. Prior research has highlighted their susceptibility to smoke in response to cues in their environment, which is stronger in people who smoke less than daily than in those who smoke daily [72]. Thus, the capacity of treatment with the SiS app to impact the ability to abstain from smoking in these circumstances is particularly promising.

Further supporting proof-of-concept evidence for the efficacy of the SiS app were the observed strong impacts of the SiS app on secondary proof-of-concept markers, which were additional therapeutic targets. These findings suggested that engaging with the SiS app also helped people who smoke less than daily experience lower levels of craving and higher levels of positive affect. These effects are consistent with the conceptual model underpinning this treatment approach, which hypothesized that regular engagement with positive psychology exercises would increase positive affect. This increased level of positive affect would then lead to a decreased desire to smoke [31]. Previously, a large laboratory cue-reactivity study with people who smoke less than daily [36], had demonstrated the potency of positive affect cues to reduce craving. Our results are in line with these findings and extend them beyond the laboratory environment. Taken together, the conceptual evidence for the efficacy of the SiS app is strong.

The observed lack of group differences between the SiS app and the frequently used and widely distributed smoking cessation tools QG and CtA on treatment acceptability indices further supports the potential impact of the novel SiS intervention. In this RCT, we assessed both content and technological satisfaction with the treatment approach. Across the 3 randomized groups, people who smoke less than daily were, by and large, equally satisfied with the smoking cessation treatment they received. In comparison to the QG app, participants were also equally satisfied with the level of technology they had to master in SiS to engage with the treatment. Given that participants in the control conditions were asked to engage with the standard of care smoking cessation materials developed and delivered by the NCI, it is quite encouraging that the SiS app was able to achieve similar levels of satisfaction. The fact that engagement with the SiS app resulted in better outcomes in terms of self-efficacy, positive affect, and craving, despite a lack of differences in likeability of the treatment, speaks to the effectiveness of the content experienced in using the SiS app.

The feasibility indices tracked as part of this RCT showed that participants meaningfully interacted with the app. Participants used the app on 33 out of 49 days of the treatment. From prior work, we know that the majority of time SiS users spent with the app is focused on completing the daily happiness exercises. For version 2 of the SiS app, 96% of the days participants used the SiS app, they completed the daily happiness exercise [39]. For version 1, app use was largely driven by completing happiness exercises, with 73% of participants completing happiness exercises on any given day [30]. Thus, it is likely that participants in this study as well spent a majority of their app time on happiness exercises. In self-report surveys, participants reported spending 35 to 40 minutes each week using the app. This length of app use time is comparable to the length of a clinician-delivered session, but it is achieved in small intervals over many days at the convenience of the participant. Completing the type of happiness exercises included in the SiS app only takes approximately 4 minutes, with demonstrable, immediate impacts on positive affect [47], and requires no scheduling or wait times. Results further suggest that people using the SiS app were more able to convert exposure to information to practice, with SiS users using more smoking cessation strategies in their daily lives. This effect was particularly strong for use of positive psychotherapy smoking cessation strategies [33] emphasized in the SiS app (Cohen *d*=0.55 SiS3 vs QG, Cohen *d*=0.42 SiS3 vs CtA), which are not typically part of smoking cessation treatment. A full-scale trial that is powered to detect differences in 30-day smoking cessation PPA is needed to test whether greater engagement in positive psychology strategies and greater self-efficacy translate to meaningful difference in smoking abstinence rates.

Regarding smoking abstinence rates, abstinence rates in our study were unusually high across all treatment groups. These higher abstinence rates are unlikely to be due to our focus on people who smoke less than daily, as prior RCTs showed low abstinence rates for people who smoke less than daily (5%-11%) [24,25]. These high abstinence rates may be due to our reliance on self-report; prior RCTs involving people who smoke less than daily used biochemical verification. However, it is important to note that the only fully powered RCT to date on the efficacy of a smartphone app for smoking cessation also relied solely on self-report and reported much lower abstinence rates (ie, 20% at the 6-month follow-up) compared to what we observed in this study [28]. Moreover, the self-reported abstinence rates in this study are in line with our previous findings in participants using prior versions of the SiS app (ie, 53% in study 1 on the SiS app, 56% in study 2) [30,39]. This leads us to believe that abstinence rates were higher in this study compared to other RCTs evaluating smoking cessation smartphone apps [28,49] because our procedures were more demanding. Unlike the largest RCT on a smoking cessation app to date [28], our study required participants to engage in an onboarding session with study staff. A smaller RCT, however, provided extensive technical support to study participants [49]. Possibly the nature of the support was more technical in nature and less focused on engaging with smoking cessation tools. In addition, our study was relatively demanding in terms of the steps participants had to take to get into the study, including the potential to not be able to proceed to enrollment if participants did not carefully read surveys. It is possible that these factors could explain the unusually high abstinence rates observed in this study. If so, it is worth investigating exactly what contributed to these high abstinence rates, as perhaps these mechanisms could be leveraged to increase therapeutic benefit. Perhaps even more puzzling is the rising abstinence rates over time, with more participants reporting abstinence at 6-month follow-up than 6-week follow-up. This increase in abstinence occurred in all 3 groups. Our best guess for this phenomenon is that our study procedures resulted in selecting participants who took their quit attempt very seriously, thereby enabling deep learning of smoking cessation strategies during this quit attempt, which may have aided them in staying quit or using these skills for a new quit attempt with greater success. They also had full access to the assigned materials throughout the duration of the study, and thus could have used all materials well beyond the “prescribed” treatment period.

### Strengths and Limitations

This study’s strengths include using 2 active, standard of care control conditions, controlling for onboarding time and attention between conditions, recruiting a national sample with racial-ethnic diversity similar to the racial-ethnic diversity of the US population of people who smoke less than daily [73], recruiting a sample comparable to people who smoke less than daily reported on in other studies, based on smoking behavior [74] and quit attempts [18], and the use of repeated measures per participant over time to increase measure sensitivity to individual differences and reliability.

This trial also had some limitations. First, this proof-of-concept trial was powered to detect differences in psychosocial constructs relevant to smoking cessation and not powered to detect differences in smoking cessation rates. Thus, the implications of this trial are limited to how well the treatment achieved differences in self-efficacy, positive affect, craving, and the use of smoking cessation strategies, and it remains to be tested how well these psychosocial constructs translate into successful smoking cessation. Second, it should be kept in mind that only a subset of those people initially interested in the study ultimately participated in the study. Thus, our study procedures may have inadvertently screened-out people less committed to making a quit attempt, thereby restricting implications of this study to people committed to making a quit attempt. Third, our onboarding procedures were more hands-on than a real-world scenario. In the real world, a person seeking smoking cessation support via smartphone app would simply browse through options in their phone’s app store, choose an app, download it, and start using it. In our study, participants did not need to choose an app. Instead, they were given clear guidance on how to use a specific app in a one-on-one phone call with clear instructions and comprehension checks. This type of onboarding is not standard. Interestingly, however, providing such guidance could be done on a larger scale through video onboarding, the help from digital navigators, or a combination of both [75,76]; this would still be shorter and require less training than telephone Quitline support. Future studies on this issue may be warranted. Such studies should examine the use of smartphone apps by people who smoke less than daily. Very little research exists regarding the type of smoking cessation support that people who smoke less than daily may accept. Existing evidence [20] suggests smartphone apps may be a particularly potent way in which to engage people who smoke less than daily in smoking cessation support, but more research is needed to demonstrate the real-world use of these technologies by this population.

It is also notable that elevated depressive symptoms varied across the three treatment groups, ranging from 38% in the SiS group to 58% in the CtA group, despite randomization. Overall, this prevalence of depressive symptoms is in line with findings on help-seeking people who smoke, where 39%-40% of callers to state quitlines reported symptoms in line with mild depression or major depression [77,78]. It is also important to note that the prevalence of nondaily smoking patterns among people who smoke is rising among those with serious mental health issues [12]. It is unclear how this difference between groups may have impacted study results. Past research has highlighted that smoking cessation may be harder for people with depressive symptoms [79,80]. At the same time, our previous findings on the same positive psychology exercises used in the SiS app have suggested that people with low positive affect benefit the most from positive psychology exercises [47]. Future research should include presence of depressive symptoms as a stratification factor to remove this ambiguous confounder from the interpretation of future results. Finally, we restricted study participation to residents of the United States. Thus, it is unclear how the findings of this study translate to findings that may be obtained when testing this treatment approach in other countries with different smoking cessation attitudes, infrastructure, and resources.

### Conclusions

The SiS app demonstrated superior performance on increasing self-efficacy to abstain from smoking and other targeted psychological constructs compared to 2 active and relevant control conditions. These superior treatment effects cannot be attributed to greater likeability of the SiS treatment approach because satisfaction ratings were largely similar across randomized groups, and thus speak to the effectiveness of the content experienced by individuals who smoke less than daily when using the SiS app. Feasibility indices showed that people who smoke less than daily interacted meaningfully with the SiS app, suggesting that they successfully translated the knowledge gained from the app into practical use in their daily lives. Together, these findings provide strong evidence for the conceptual underpinnings of the SiS app and provide a good rationale for conducting a large-scale RCT that can test the effectiveness of the SiS app on smoking cessation.

## Appendix

Multimedia Appendix 1 Supplementary tables of study outcomes.

Multimedia Appendix 2 CONSORT-eHEALTH checklist (V 1.6.1).

## Abbreviations

Brief-QSU: Brief Questionnaire of Smoking Urges
CSQ: Client Satisfaction Questionnaire
CtA: Clearing the Air
DCB-SF: Decisional Balance Inventory for Smoking short form
NCI: National Cancer Institute
PPA: point prevalence abstinence
QG: QuitGuide
RCT: randomized controlled trial
REDCap: Research Electronic Data Capture
SiS: Smiling instead of Smoking
SUS: System Usability Scale

## Data Availability

The datasets analyzed during this study are available from the corresponding author on reasonable request.
