# Supplementary material for: Smoking Cessation Smartphone App for Nondaily Smoking With Telephone Onboarding: Proof-of-Concept Randomized Controlled Trial
Source: JMIR Mhealth Uhealth. 2025 Jan 15;13:e53971. doi: 10.2196/53971 (PMC11780296; doi:10.2196/53971)
Supplement: Multimedia Appendix 1 [file mhealth_v13i1e53971_app1.docx]

**Supplementary Online Content**

This is a Multimedia Appendix to the following full manuscript published in JMIR mHealth and uHealth:

Hoeppner BB, Siegel KR, Futter AE, Finley-Abboud D, Williamson AC, Kahler CW, Park ER, Hoeppner SS. Results of a of a smoking cessation smartphone app for nondaily smoking with telephone onboarding: A proof-of-concept randomized controlled trial. 2004. JMIR mHealth and uHealth. DOI: https://doi.org/10.2196/53971

For full copyright and citation information see [the](http://dx.doi.org/10.2196/jmir.53971) main manuscript.

1. **Table S1.** Generalized linear mixed model effects for primary, secondary, and exploratory study outcomes that met assumptions for parametric analysis.
2. **Table S2.** End-of-treatment (week 6) group differences from generalized linear mixed models for primary, secondary, and exploratory study outcomes that met assumptions for parametric analysis.
3. **Table S3.** Wilcoxon rank sum tests (Mann-Whitney U tests) of pairwise group differences for study outcomes that were not normally distributed.
4. **Literature Cited.**

This supplementary material has been provided by the authors to give readers additional information about their work.

**Table S1.** Generalized linear mixed model effects for primary, secondary, and exploratory study outcomes that met assumptions for parametric analysis.

|  |  |  |  | **Group** | | | |  | **Week** | | | |  | **Group*Week** | | | |
| --- | --- | --- | --- | --- | --- | --- | --- | --- | --- | --- | --- | --- | --- | --- | --- | --- | --- |
|  |  |  |  | **N DF** | **Den DF** | **F value** | **Pr > F** |  | **N DF** | **Den DF** | **F value** | **Pr > F** |  | **N DF** | **Den DF** | **F value** | **Pr > F** |
| Primary Outcome | | | | | | | | |  |  |  |  |  |  |  |  |  |
|  |  | Self-efficacy (SEQ-12) - overall | | 2 | 214 | 4.01 | 0.020 |  | 4 | 188 | 31.25 | <.001 |  | 8 | 265 | 0.72 | 0.676 |
|  |  | Self-efficacy (SEQ-12) - internal | | 2 | 215 | 3.25 | 0.041 |  | 4 | 187 | 32.65 | <.001 |  | 8 | 265 | 1.08 | 0.378 |
|  |  | Self-efficacy (SEQ-12) - external | | 2 | 214 | 3.92 | 0.021 |  | 4 | 190 | 24.23 | <.001 |  | 8 | 267 | 0.49 | 0.862 |
| Secondary Outcomes | | | | | | | | |  |  |  |  |  |  |  |  |  |
|  | *Treatment Acceptability* | | |  |  |  |  |  |  |  |  |  |  |  |  |  |  |
|  |  | Client satisfaction (CSQ-8) | | 2 | 192 | 1.01 | 0.365 |  |  |  | n/a |  |  |  |  | n/a |  |
|  |  | System usability (SUS) | | 1 | 126 | 0.69 | 0.408 |  |  |  | n/a |  |  |  |  | n/a |  |
|  |  | App likability rating | | 1 | 127 | 0.45 | 0.502 |  |  |  | n/a |  |  |  |  | n/a |  |
|  |  | App satisfaction rating | | 1 | 126 | 0.64 | 0.425 |  |  |  | n/a |  |  |  |  | n/a |  |
|  | *Treatment Feasibility* | | |  |  |  |  |  |  |  |  |  |  |  |  |  |  |
|  |  | Use of smoking cessation strat. | | 2 | 186 | 2.18 | 0.116 |  |  |  | n/a |  |  |  |  | n/a |  |
|  |  | Use of positive psychology strat. | | 2 | 186 | 5.20 | 0.006 |  |  |  | n/a |  |  |  |  | n/a |  |
|  |  | Perceived impact on quitting | | 2 | 190 | 2.99 | 0.053 |  |  |  | n/a |  |  |  |  | n/a |  |
| Exploratory outcomes (i.e., secondary proof-of-concept efficacy outcomes) | | | | | | | | | | | | | | | | | |
|  | *Positive affect* | | |  |  |  |  |  |  |  |  |  |  |  |  |  |  |
|  |  | Positive affect (PANAS) | | 2 | 219 | 2.26 | 0.107 |  | 4 | 186 | 10.23 | <.001 |  | 8 | 262 | 1.01 | 0.431 |
|  | *Desire to smoke* | | |  |  |  |  |  |  |  |  |  |  |  |  |  |  |
|  |  | Craving (Brief QSU) | | 2 | 203 | 3.00 | 0.052 |  | 4 | 186 | 31.49 | <.001 |  | 8 | 262 | 2.08 | 0.038 |
|  | *Attitudes toward smoking* | | |  |  |  |  |  |  |  |  |  |  |  |  |  |  |
|  |  | Adverse effects (ATS) | | 2 | 215 | 0.37 | 0.692 |  | 4 | 176 | 8.62 | <.001 |  | 8 | 250 | 0.75 | 0.649 |
|  |  | Psychoactive benefits (ATS) | | 2 | 216 | 0.69 | 0.505 |  | 4 | 181 | 41.21 | <.001 |  | 8 | 256 | 1.83 | 0.073 |
|  |  | Pleasure (ATS) | | 2 | 213 | 1.78 | 0.171 |  | 4 | 179 | 34.09 | <.001 |  | 8 | 254 | 0.78 | 0.617 |
|  |  | Positive expectancies (DCB-SF) | | 2 | 216 | 1.45 | 0.236 |  | 4 | 182 | 31.60 | <.001 |  | 8 | 258 | 1.10 | 0.366 |
|  |  | Negative expectancies (DCB-SF) | | 2 | 219 | 0.90 | 0.407 |  | 4 | 185 | 3.35 | 0.011 |  | 8 | 262 | 1.08 | 0.380 |

Notes: N DF = denominator degrees of freedom; Den DF = denominator degrees of freedom; Pr>F = p-value of significance test for the indicated overall effect in the GLMM; degrees of freedom were based on the degrees of freedom calculations detailed by Kenward and Roger (1997). SiS3 = Version 3 of the SiS app; QG = National Cancer Institute (NCI) Quit Guide app; CtA = NCI "Clearing the Air" brochure; SEQ-12 = Smoking Self-Efficacy Questionnaire; CSQ-8 = Client Satisfaction Scale; SUS = System Usability Scale; strat. = strategies; PANAS = Positive and Negative Affect Schedule; Brief QSU = Brief Questionnaire of Smoking Urges; ATS = Attitudes Towards Smoking scale; DCB-SF = Decisional Balance Inventory for Smoking Short Form.

**Table S2.** End-of-treatment (week 6) group differences from generalized linear mixed models for primary, secondary, and exploratory study outcomes that met assumptions for parametric analysis.

|  |  |  |  | **Effect contrast: SiS3 vs. QG at week 6** | | | |  | **Effect contrast: SiS3 vs. CtA at week 6** | | | |
| --- | --- | --- | --- | --- | --- | --- | --- | --- | --- | --- | --- | --- |
|  |  |  |  |  |  |  |  |  |  |  |  |  |
|  |  |  |  | **Est.** | **95% CI** | | **Pr > F** |  | **Est.** | **95% CI** | | **Pr > F** |
| Primary Outcome | | | |  |  |  |  |  |  |  |  |  |
|  |  | Self-efficacy (SEQ-12) - overall | | 7.33 | [1.07, | 13.59] | 0.0219 |  | 8.78 | [2.41, | 15.15] | 0.0072 |
|  |  | Self-efficacy (SEQ-12) - internal | | 7.62 | [0.65, | 14.59] | 0.0324 |  | 8.92 | [1.82, | 16.02] | 0.0141 |
|  |  | Self-efficacy (SEQ-12) - external | | 7.26 | [0.75, | 13.78] | 0.0291 |  | 8.90 | [2.25, | 15.54] | 0.0089 |
| Secondary Outcomes | | | | | | | | |  |  |  |  |
|  | *Treatment Acceptability* | | |  |  |  |  |  |  |  |  |  |
|  |  | Client satisfaction (CSQ-8) | | 0.29 | [-1.50, | 2.09] | 0.7469 |  | 1.26 | [-0.56, | 3.08] | 0.1747 |
|  |  | System usability (SUS) | | -2.43 | [-8.22, | 3.36] | 0.4078 |  | n/a | | | |
|  |  | App likability rating | | 0.11 | [-0.21, | 0.44] | 0.5018 |  | n/a | | | |
|  |  | App satisfaction rating | | 0.14 | [-0.21, | 0.49] | 0.4246 |  | n/a | | | |
|  | *Treatment Feasibility* | | |  |  |  |  |  |  |  |  |  |
|  |  | Use of smoking cessation strategies | | 0.23 | [0.01, | 0.45] | 0.0429 |  | 0.16 | [-0.06, | 0.39] | 0.1600 |
|  |  | Use of positive psychology strategies | | 0.31 | [0.11, | 0.50] | 0.0025 |  | 0.24 | [0.04, | 0.44] | 0.0174 |
|  |  | Perceived impact on quitting | | 0.15 | [-0.08, | 0.38] | 0.1996 |  | 0.29 | [0.06, | 0.53] | 0.0154 |
| Exploratory outcomes (i.e., secondary proof-of-concept efficacy outcomes) | | | | | | | | |  |  |  |  |
|  | *Positive affect* | | |  |  |  |  |  |  |  |  |  |
|  |  | Positive affect (PANAS) | | 3.65 | [0.81, | 6.50] | 0.0121 |  | 2.86 | [-0.02, | 5.75] | 0.0514 |
|  | *Desire to smoke* | | |  |  |  |  |  |  |  |  |  |
|  |  | Craving (Brief QSU) | | -4.50 | [-7.64, | -1.35] | 0.0053 |  | -4.56 | [-7.75, | -1.37] | 0.0053 |
|  | *Attitudes toward smoking* | | |  |  |  |  |  |  |  |  |  |
|  |  | Adverse effects (ATS) | | 0.04 | [-0.17, | 0.24] | 0.7382 |  | 0.05 | [-0.16, | 0.26] | 0.6496 |
|  |  | Psychoactive benefits (ATS) | | -0.24 | [-0.58, | 0.10] | 0.1710 |  | -0.29 | [-0.64, | 0.06] | 0.0997 |
|  |  | Pleasure (ATS) | | -0.13 | [-0.46, | 0.20] | 0.4418 |  | -0.35 | [-0.68, | -0.02] | 0.0392 |
|  |  | Positive expectancies (DCB-SF) | | -6.76 | [-15.38, | 1.85] | 0.1231 |  | -7.91 | [-16.65, | 0.82] | 0.0755 |
|  |  | Negative expectancies (DCB-SF) | | -2.08 | [-10.59, | 6.43] | 0.6302 |  | 2.67 | [-5.95, | 11.29] | 0.5420 |

Notes: P>F = p-value of significance test for pairwise group-comparisons; SiS3 = Version 3 of the SiS app; QG = National Cancer Institute (NCI) Quit Guide app; CtA = NCI "Clearing the Air" brochure; SEQ-12 = Smoking Self-Efficacy Questionnaire (range 0-100, where higher scores indicate greater self-efficacy to abstain from smoking); CSQ-8 = Client Satisfaction Scale (range: 0-27, where higher scores indicate higher client satisfaction); SUS = System Usability Scale (range: 0-100, where higher scores mean greater app usability); PANAS = Positive and Negative Affect Schedule (range 10 to 50, where higher scores indicate higher levels of positive affect); Brief QSU = Brief Questionnaire of Smoking Urges (range: 7 to 70, with higher scores indicating greater cigarette craving); ATS = Attitudes Towards Smoking scale (sub-scale mean score range: 1 to 5, where higher scores indicate stronger agreement with the subscale statements); DCB-SF = Decisional Balance Inventory for Smoking Short Form (sub-scale range: 0-100, where higher scores indicate greater agreement with the pros and cons of smoking, respectively).

**Table S2 (continued).** End-of-treatment (week 6) group differences from generalized linear mixed models for primary, secondary, and exploratory study outcomes that met assumptions for parametric analysis.

|  |  |  |  | **Effect contrast: QG vs. CtA at week 6** | | | |
| --- | --- | --- | --- | --- | --- | --- | --- |
|  |  |  |  |  |  |  |  |
|  |  |  |  | **Est.** | **95% CI** | | **Pr > F** |
| Primary Outcome | | | |  |  |  |  |
|  |  | Self-efficacy (SEQ-12) - overall | | 1.44 | [-4.94, | 7.83] | 0.6561 |
|  |  | Self-efficacy (SEQ-12) - internal | | 1.31 | [-5.79, | 8.40] | 0.7172 |
|  |  | Self-efficacy (SEQ-12) - external | | 1.63 | [-5.00, | 8.27] | 0.6277 |
| Secondary Outcomes | | | |  |  |  |  |
|  | *Treatment Acceptability* | | |  |  |  |  |
|  |  | Client satisfaction (CSQ-8) | | 0.96 | [-0.84, | 2.77] | 0.2942 |
|  |  | System usability (SUS) | | n/a | | | |
|  |  | App likability rating | | n/a | | | |
|  |  | App satisfaction rating | | n/a | | | |
|  | *Treatment Feasibility* | | |  |  |  |  |
|  |  | Use of smoking cessation strategies | | -0.07 | [-0.29, | 0.16] | 0.5491 |
|  |  | Use of positive psychology strategies | | -0.06 | [-0.26, | 0.14] | 0.5326 |
|  |  | Perceived impact on quitting | | 0.14 | [-0.09, | 0.37] | 0.2390 |
| Exploratory outcomes (i.e., secondary proof-of-concept efficacy outcomes) | | | |  |  |  |  |
|  | *Positive affect* | | |  |  |  |  |
|  |  | Positive affect (PANAS) | | -0.79 | [-3.68, | 2.10] | 0.5914 |
|  | *Desire to smoke* | | |  |  |  |  |
|  |  | Craving (Brief QSU) | | -0.07 | [-3.24, | 3.11] | 0.9670 |
|  | *Attitudes toward smoking* | | |  |  |  |  |
|  |  | Adverse effects (ATS) | | 0.01 | [-0.20, | 0.22] | 0.9012 |
|  |  | Psychoactive benefits (ATS) | | -0.05 | [-0.40, | 0.30] | 0.7663 |
|  |  | Pleasure (ATS) | | -0.22 | [-0.56, | 0.11] | 0.1900 |
|  |  | Positive expectancies (DCB-SF) | | -1.15 | [-9.87, | 7.57] | 0.7950 |
|  |  | Negative expectancies (DCB-SF) | | 4.75 | [-3.83, | 13.33] | 0.2761 |

Notes: P>F = p-value of significance test for pairwise group-comparisons; SiS3 = Version 3 of the SiS app; QG = National Cancer Institute (NCI) Quit Guide app; CtA = NCI "Clearing the Air" brochure; SEQ-12 = Smoking Self-Efficacy Questionnaire (range 0-100, where higher scores indicate greater self-efficacy to abstain from smoking); CSQ-8 = Client Satisfaction Scale (range: 0-27, where higher scores indicate higher client satisfaction); SUS = System Usability Scale (range: 0-100, where higher scores mean greater app usability); PANAS = Positive and Negative Affect Schedule (range 10 to 50, where higher scores indicate higher levels of positive affect); Brief QSU = Brief Questionnaire of Smoking Urges (range: 7 to 70, with higher scores indicating greater cigarette craving); ATS = Attitudes Towards Smoking scale (sub-scale mean score range: 1 to 5, where higher scores indicate stronger agreement with the subscale statements); DCB-SF = Decisional Balance Inventory for Smoking Short Form (sub-scale range: 0-100, where higher scores indicate greater agreement with the pros and cons of smoking, respectively).

**Table S3.** Wilcoxon rank sum tests (Mann-Whitney U tests) of pairwise group differences for study outcomes that were not normally distributed.

|  |  |  |  | **Pairwise model: SiS3 vs. QG** | |  | **Pairwise model: SiS3 vs. CtA** | |  | **Pairwise model: QG vs. CtA** | |  |
| --- | --- | --- | --- | --- | --- | --- | --- | --- | --- | --- | --- | --- |
|  |  |  |  |  |  |  |  |  |  |  |  |  |
|  |  |  |  | **Wilcoxon Z** | **Pr > \|Z\|** |  | **Wilcoxon Z** | **Pr > \|Z\|** |  | **Wilcoxon Z** | **Pr > \|Z\|** |  |
| Secondary Outcomes | | | | | | |  |  |  |  |  |  |
|  | *Treatment Feasibility* | | |  |  |  |  |  |  |  |  |  |
|  |  | Time applying content (min./wk.) | | 1.93 | 0.1315 |  | 1.40 | 0.3403 |  | -0.38 | 0.9241 |  |
|  |  | App usage (# of days used) | | 0.81 | 0.4182 |  | n/a |  |  | n/a |  |  |
|  |  | App use - weeks 1-3 (mins./wk.) | | 2.68 | 0.0073 |  | n/a |  |  | n/a |  |  |
|  |  | App use - weeks 4-7 (mins./wk.) | | 1.91 | 0.0563 |  | n/a |  |  | n/a |  |  |
| Exploratory outcomes (i.e., secondary proof-of-concept efficacy outcomes) | | | | | | |  |  |  |  |  |  |
|  | *Treatment effectiveness* | | |  |  |  |  |  |  |  |  |  |
|  |  | Change in past week cigs. (cigs./wk.) | | 0.40 | 0.9152 |  | 1.37 | 0.3557 |  | 1.17 | 0.4692 |  |

Notes: SiS3 = Version 3 of the SiS app; QG = National Cancer Institute (NCI) Quit Guide app; CtA = NCI "Clearing the Air" brochure; mins. = minutes; cigs. = cigarettes; wk = week. For models with all three groups (i.e., time applying content and change in past cigarettes), we used the Dwass, Steel, Critchlow-Fligner (DSCF) multiple comparison analysis, which is based on pairwise two-sample Wilcoxon comparisons (Critchlow and Fligner, 1991).

**Literature cited**

Critchlow, D. E., & Fligner, M. A. (1991, 1991/01/01). On distribution-free multiple comparisons in the one-way analysis of variance. Communications in Statistics - Theory and Methods, 20(1), 127-139. https://doi.org/10.1080/03610929108830487

Kenward, M. G., & Roger, J. H. (1997). Small sample inference for fixed effects from restricted maximum likelihood. Biometrics, 53(3), 983-997. https://doi.org/10.2307/2533558
